# Case of Aneurism from a Puncture of the Humoral Artery, Cured by Pressure above the Injury, in a Letter from Joseph Adams, M. D. &c. &c. of Madeira

**Published:** 1801-12

**Authors:** 


### *Case of Aneurism from a Puncture of the humoral Artery, cured by Pressure above the Injury, in a Letter from* Joseph Adams, M. D. *&c. &c. of Madeira.*

In the latter end of the year, 1796, a young Baronet was recommended to this island on account of pulmonary complaints, threatening consumption. On the 4th of February, 1797, he applied to me, having a return of some of the symptoms. He had been in the habit of being bled frequently, and the present symptoms indicated that remedy. In this island, when a physician orders blood-letting, the custom is to apply to a barber. As this is very repugnant to our English habits, it seemed to savor of affectation not to offer my services, especially as it appeared the wish of the family at whose house my patient resided.

Observing a cicatrix on the vena mediana basilica, I chose it for the present operation. The slight resistance made to the lancet was imputed to the callosity of the cicatrix. The blood started with considerable force; but not with greater than frequently attends venæsection in warm weather: there was no appearance of saltus. The bleeding was stopped with some difficulty, but this seemed readily accounted for by a tight flannel sleeve which we had neglected to take off or rip open. In the evening the orifice bled a-fresh, but was stopped before my arrival. On the following morning considerable extravasation of blood appeared in the arm, which was imputed to the laxity of the cellular membrane in this subject. The orifice was tender, and did not heal readily. In a few days, however, it was thought so much better as to render any further attendance unnecessary.

It ought here to be remarked, that my patient was a man of that native politeness which made him feel for the embarrassment of another. Conscious, therefore, that a subject of this kind must be unpleasant to the operator, he always endeavoured to make the least of it; and to this, among other causes, we may attribute the succeeding events.

Sir B. now returned to his usual exercises, which I encouraged as a means of promoting absorption, and still more on account of his pulmonary complaints. His health so far returned as to render my attendance unnecessary; and when we met, which in this small circle could not but be frequently, he always assured me his arm was very well. In this situation things remained till the middle of March, when, meeting at a dinner party, he requested to see me the following morning.

I then learned that the arm had never recovered its original

 size,

ſize, and that a day or two before a ſmall tumour had ariſen. It was lower in the arm than the cicatrix, and on preſſure a very obſcure pulſation might be perceived: it was firmly bound by the faſcia, and not at all diſcoloured or painful. In this ſtage it was not eaſy to diſcover whether an abſceſs was forming, from a ſmall portion of blood remaining unabſorbed and loſing its life; or whether, what was moſt to be dreaded, an artery had been wounded. In the latter caſe there was a fair preſumption, at leaſt, that the wound might be through a vein ſo as to form the varicoſe aneuriſm. At all events, if the caſe was true aneuriſm, there could now be no other remedy but the operation; and as the inciſion had been made ſix weeks, there was too much reaſon to ſuppoſe that the artery muſt be dilated beyond the orifice. If, therefore, the operation ſhould be performed higher up, according to the improved practice, no inconvenience could ariſe from deferring it for the preſent.

In this ſituation things remained till the 20th of March, when my patient made a tour of a few days into the country. On his return the tumour ſeemed increaſed; but as that was doubtful, a meaſure of the arm was taken to aſcertain its future progreſs. For a fortnight it remained ſtationary, when it ſuddenly increaſed with the ſame circumſcribed appearance, and an evident ſurrounding diffuſed extravaſation. A ſtrong preſſure was now applied on the upper part of the artery where it riſes ſuperficially. From the lax ſtate of the cellular membrane the veſſel could be ſo preſſed againſt the bone as conſiderably to leſſen its pulſation. In order to protect the circulation in the reſt of the limb, pieces of cane, armed with linen, were placed under different parts of the bandage to keep it hollow. By theſe means there was reaſon to expect that the diameter of the artery might be leſſened, and its collateral branches increaſed, which would very much leſſen the future inconveniences of an operation. Nor was I without hopes, as my patient found no inconvenience from ſtrong preſſure, that it might be gradually increaſed ſo as to obliterate the artery altogether. But either from the difficulty of keeping the bandage fixed, or becauſe the degree of preſſure was unequal to the intended object, in about ten days we had an increaſe of tumour, attended with all the former appearances, and for the firſt time with a ſlight degree of pain.

It now became neceſſary to take ſome decided ſtep, and as the high operation was in ſome reſpects new, I could not but be anxious to have the aſſiſtance of a ſurgeon who had recently left the buſy ſcenes of improvement and practice. His Majeſty's ſhip the Terpſichore, at that time appearing in our roads, I called the ſurgeon of it, and the only Britiſh phyſician in the iſland, to to my affiftance; but had the misfortune to ftand alone in my propofal of either performing the operation higher up in the arm, by a fingle ligature, or trying the effect of preffure only.* Some other difficulties which occurred, determined me to proceed with my patient to the fleet before Cadiz, in order to procure the beft affiftance circumftances would permit.

Finding he had felt fo little inconvenience from preffure on a former occafion, I refolved to try the ufe of the tourniquet, keeping the fillet hollow by circular pieces of wood under it in different parts. It was hardly credible how little uneafinefs this was attended with, though the preffure was fo confiderable as almoft to ftop all pulfation at the wrift, and even to occafion fome oedema in the hand. This laft circumftance made me anxious for fome apparatus that might prefs more immediately upon the artery, and leave the reft of the limb as free as poffible. On enquiry, the furgeon of the frigate recollected a fmall inguinal trufs, which was not in ufe. This was directed to be cut fomewhat fhorter, and to be bent to a fmaller angle. The bulb was fixed about the middle of the humerus, immediately over the artery, which it preffed fo clofe to the bone as to prevent all pulfation in the tumour. My patient, however, felt no uneafinefs; and though on feeling the hand it was fenfibly colder than the other, he perceived no numbnefs nor fenfe of cold. The trufs was fixed on about four in the afternoon, and remained fo till between nine and ten, when it was the cuftom of the cabin to retire to reft. The inftrument was ftill left on; but as the arm refted on the bed on one fide, and had the preffure of the cloths on the other, and alfo as there was no longer the roughnefs of the woolen coat to refift the leather, it was not probable the apparatus fhould long remain fteady. About midnight I was awakened from my illufive expectations of fuccefs to hear my patient, for the firft time, complain of extreme pain. I found the inftrument fo far from preffing on the artery that it feemed fcarcely to make any preffure at all. The tumour was fo perfectly folid that there could be no doubt the blood had coagulated. There was no pain near the part preffed upon, nor any where higher in the limb than the aneurifm, but immediately under and below it. If this arofe from the preffure of the folid

* The following is Mr. Home's defcription of the operation in the ham: Tranfaction of a Society, &c. page 176.—Such is the operation in the true popliteal aneurifm. In the cafe of the arm, if the parts are lefs complicated yet the difficulty is more than increafed, becaufe there is no other fack than confolidated cellular membrane. I once faw a man expire under the operation for the popliteal aneurifm, performed in the old way by Mr. Martin, in St. Thomas's Hofpital.

folid coagulum, which the patient defcribed as hard as a brick-bat, the only remedy appeared by warm fomentations to relax the parts as much as poffible. Thefe were continued for an hour and a half before the pain entirely fubfided, though there were frequent intermiffions. After this the patient refted till day light. In the morning he was fomewhat feverifh, with occafional but flight returns of pain, chiefly in the fore-arm. The whole arm was thickened from the part to which the preffure had been applied to the wrift, and two of the fingers were infenfible. About nine he arofe, and during the reft of the day had frequent returns of what he ftiled tearing and rending pains in the fore-arm and wrift. At night an obfcure pulfation was perceived, the aneurifmal tumour confiderably increafed, and the patient complained much of its weight.

The following morning the tumour remained of the fame fize; but the fkin was florid, tranfparent, and about the centre purplifh, intermixed with parts of a paler hue. I was now more alarmed than before. The fingers remaining infenfible, and the pulfe at the wrift very obfcure, there was reafon to believe that if the artery had recovered itfelf, it was only as low as the tumour: hence it was impoffible to calculate what might be the confequence of the whole force of the heart and arteries urging the blood againft a part which feemed already giving way.

As, however, preffure had fucceeded in obliterating part of the artery, there was every thing to hope from a more regular continuance of it. The thickening of the arm rendered it difficult to fix the trufs in fuch a manner as to prefs fufficiently againft the bone. A part higher up was therefore preferred, not more than two inches from the axilla, the trufs was bent to a ftill fmaller angle, and a number of pieces of wood, of a wedge fhape, were prepared to place under the fmaller end, in order to increafe its preffure *ad libitum*. Under the thick end was placed a fmall fquare piece of wood, formed like that of the tourniquet. The fmaller end preffing on the artery, a groove was made on the broader and upper part to receive the edge of the trufs. That no time might be loft, the apparatus was applied as foon in the morning as my patient could be placed on the fofa, with his arm extended on a ftool.

After about an hour he complained of intenfe pain between the tumour and the part to which the preffure was applied. On examination I found, that in fpite of all my endeavours the artery had recovered itfelf, and this pain feemed to arife either from the blood again diftending the contracted part of the artery, or from the veffel itfelf attempting to dilate, notwithftanding the refiftance of the trufs. This appeared the more certain, certain, becaufe a pulfation, not very obfcure, could be felt below the preffure; and becaufe, on the application of my finger immediately over the artery, the pain ceafed. The apparatus was placed on a frefh and thicker wedge at the fmall end of the trufs; but in the fpace of half an hour the pain returned, and was eafily relieved by elevating the narrow end of the trufs fo as to increafe the preffure. As my patient found immediate relief from this, he expreffed no longer any wifh to have the trufs removed. For about fix hours the pain recurred at intervals, though in a flighter degree, and was always removed by the fame means. The laft paroxyfm was whilft at dinner. After this he felt fo great a drowfinefs come over him that it was difficult to keep awake, though he continued to eat. It feems at leaft probable, that the blood ceafing altogether to flow through the artery, produced, for a fhort time, a fulnefs in the veffels about the brain fufficient to occafion this heavinefs. In the night he had pains in his hand and fore-arm, but flept tolerably well. In the morning the arm appeared thickened from the part preffed downwards. All the fingers and thumb were infenfible, though warmer to the feel than thofe of the other hand, thickened, and of a florid complexion. A pain was felt alternately at the back of the hand, and the infide of the fore-arm; and the veins of both, whenever the pain occurred, were particularly turgid. Cold faturnine applications feemed to give fome relief, but elevating the hand ftill more.

The occafional exacerbations of pain continued without violence, and with lefs frequency for three days, after which they never returned. There was, however, as the patient expreffed it, a fidgety kind of a feel about the hand for fome days after; and it was curious to obferve, as the thickening* of the arm gradually fubfided, fo as to expofe the more fuperficial veins, how much they were contracted in their diameter.

I have defcribed the pain, from the time the artery was clofed, as feated altogether in the veins. It is extremely difficult to reafon from a fingle inftance, but a conftant attention to every the moft minute particular, may allow a larger latitude than the common courfe of practice. From this I fhould not fcruple to fay, that the pain arofe from the valves of the veins being overftretched by a column of blood falling back fuddenly upon them. Though it is well-known that every part of the vafcular fyftem contracts its diameter in proportion to its contents, yet it is not lefs

* I have called it thickening becaufe it had not the true leucophlegmatic or œdematous appearance, retaining no impreffion of the finger, and the colour being brighter.

lefs certain that the veins, befides that they are more numerous, do not poffefs this contractile power equal to the arteries. Hence the fudden deprivation of fuch a portion of blood as the humeral artery affords, muft produce a deficiency firft in the extreme branches of the veins; in confequence of which, the valves not being fupported, a column of blood would be likely to fall fo fuddenly upon them as to produce intenfe pain from the novelty and fuddennefs of the fenfation. That this is really the cafe I had a remarkable illuftration in an aneurifm of the temporal artery, which the polite attention of the medical gentlemen of the Englifh Naval Hofpital at Lifbon, gave me an opportunity of examining.

This cafe was of two years ftanding; and, from the obftinacy of the patient, no means had been ufed for his relief. The whole artery was dilated from the temples on each fide over the cranium. The man was fubject to violent paroxyfms of pain, which he felt not in the artery, but immediately above the nofe where the veins are fuperficial. Without, however, infifting on this caufe of the pain, the fact itfelf, ftrengthened by the obfervations of others, fhows that there is frequently a kind of fympathy between arteries and their correfponding veins.

In the firft cafe related by Mr. Home of popliteal aneurifm, the femoral vein was found obliterated. In the fecond cafe, immediately after the operation, the fuperficial veins of the leg became turgid. In this operation the vein was included in the ligature, but thofe remaining were more than fufficient for whatever blood could be conveyed after the deftruction of the artery. Morgaffie furnifhes inftances much more ftriking than thefe: I need only refer you to XL, 23. Thofe of VIII. 11, and XLIII, 22, may be afcribed to a gradual enlargement in the artery, which produced a neceffity of the fame in the veins. Still, however, the exact correfpondence is worthy remarking. But the moft ftriking cafe is that related by Haller, in his Pathological Enquiries, XIX. Here the left carotid artery and jugular vein were both filled with a white, foft, and firm fubftance. In XX (note) we are referred to a cafe of aneurifm of the aorta, in which the vena cava was filled with a fat medullary fubftance. Thefe hints are, however, only thrown out for your future obfervation in cafes that are fo frequently offering themfelves to you.

That I might as little as poffible interrupt the hiftory of the fymptoms from preffure, I have taken no notice of the tumour fince the evening after the firft application of the trufs. The fkin grew more tranfparent, and difcovered under it a blacker appearance. In about two days the cuticle peeled off, and a black coagulum appeared covered with a thin pellicle, either rete rete mucosum, or stratum of coagulable lymph. The smell was that of putrid animal matter. On the following day a large quantity of serum, tinged with red particles, was discharged; the coagulum was extremely putrid, and had all the appearance of mortified integuments. I was not, however, at all alarmed at this appearance. It required no superior sagacity in one who had traced the whole progress of the mischief, to see that the coagulated blood which had retained its life, and probably taken vessels to support that life, as long as it could be useful in closing the orifice of the artery, had now lost its life, and being too considerable to be absorbed, was to be thrown out like any other extraneous body.* For three days the aperture of the skin continued to enlarge, and at the lower edge had a sloughy appearance. In every other part it was easy to see that the putrid coagulum was totally distinct from the integuments. The appearance was, however, so formidable as to alarm the surgeon of the frigate; and three days after, when we arrived at the fleet, and the case had become still more marked by a hollowness, without suppuration between the coagulum and integuments, the Surgeon-general could not be convinced that the whole was not mortification of the original parts. On the day following, the Surgeon-general of the Naval Hospital at Gibraltar, a gentleman who, by many years residence at a hospital in the metropolis, had enjoyed peculiar advantages, expressed his doubts. I mention this not out of disrespect to these gentlemen, but that the medical reader may have a just conception of the appearance; and, in case of a similar occurrence, may feel as little embarrassed as possible. It is but justice to add, that both these gentleman had the candour to acknowledge themselves convinced, and to congratulate me on having saved my patient the danger of a painful and uncertain operation.

The aperture continued to enlarge to a circle, the diameter of which could not be less than two inches, after which it became stationary. The discharge continued of the same complexion and quantity for three days; after which it became limpid, but for three days more in as great a quantity as ever. From that time it began to lessen, the putrid coagulum to be elevated, and gradually thrown out, the œdema in the arm to

* See two cases in the Edinburgh Medical Essays Abridged, vol. ii, page 53 and 55. In the first of these, the above appearances having occurred in a more recent state, are very accurately described as a "polypus substance and blood in a fluid state." I might also remark, that in this case "the hand, during the cure, was *apt* to be œdematous;" and that this œdema appears, by the account, to have always subsided suddenly. It was, therefore, probably, only a turgescence of the veins.

difappear, and the orifice to contract. By degrees a rednefs was difcovered at the bottom which looked like granulation, but was, in fact, only the mufcles expofed by the removal of the coagulum. During this whole time no applications were ufed but dry lint; the fore never was painful nor troublefome after the ferous difcharge ceafed. The fingers remained infenfible and ufelefs for feveral days; after which the joint neareft the hand acquired fome motion; and in about three weeks after the fuccefsful application of preffure, all but the thumb and fore-finger had acquired motion. In the mean while the fuperficial veins began to enlarge, an obfcure pulfation could be perceived at the wrift, and it was curious to obferve a circumfcribed line in each of the nails which divided the extreme part of a dufky hue from that nearer the root, which had the fplendour and femi-tranfparency of living nails.

It was full three weeks more before the forefinger and thumb recovered themfelves at all; even then the limb was weaker than the other, and colder to the touch. It was, however, equal to all the common purpofes of life; and when we parted at the Tagus, no difficulty was found in managing the full decanter without the help of the other hand. This was on the 11th of July. By letter of his own writing, dated January, 1798, it appears, that even then the firft joints of the thumb, fore-finger, and mid-finger had not acquired their original ftrength. "Thefe inconveniences," he adds, "are fo trifling, that I fhould not have mentioned them had you not defired me to be very particular." The fore had completely cicatrized before the conclufion of the month of July, 1797.

The cafe above related having arifen from one of the moft common operations in furgery, it is of fome importance to trace the caufes that might contribute to it, and alfo the manner of relief. The preffure of the flannel waiftcoat, mentioned at the beginning of the paper, probably obfcured the pulfation of the artery fo as to prevent its being felt before the operation. To the fame caufe may be imputed the want of force and of faltus in the flow of the blood after the incifion. This is not meant as an apology, but a confeffion; becaufe, without doubt, every part of the arm fhould have been examined, and the fleeve have been opened to prevent any embarraffment in the operation. I have before remarked, that an old cicatrix was chofen, on which the patient, to ufe his own language, had been bled a hundred times before. This occafional alteration in the courfe of the veffels is too well known to require any comment. The attempt to ftop the blood by preffure, below the orifice, did not fucceed: but the uncertain divifion of the veins at this part, often occafions this difficulty. When preffure was applied plied immediately over the orifice, the bleeding was ftopped much more eafily than often happens from venæfection in hot weather. Thefe are the only extenuating circumftances to be offered for not difcovering the error at firft. Had it been otherwife, there is no reafon to doubt but a competent preffure over the orifice, and above it, might have prevented all the fubfequent mifchief.

In the fecond ftage of the difeafe, fix weeks after the accident, the artery might as well have been obliterated as at any fubfequent period, by which means the extravafated coagulum might have been abforbed, and the inconveniences of a large fore avoided. But it will at once occur to the medical reader, that the obliteration of a large artery is not an experiment to be made on conjecture. I have already traced my doubts on that fubject; and though men, who have feen little, and write much, can readily give an unembarraffed opinion; yet, to me, it is fufficient, that one who knew too well the value of truth to fport with it, is not afhamed of expreffing his doubts on ecchymofis.

" It has often," he fays, " the appearance of incyfted tumour, but being an immediate confequence of fome accident on the parts, its nature becomes readily underftood; though, fometimes, from its fituation, it has the fymptoms of aneurifm; neither does the caufe of it contradict the idea.

" If formed over a large artery, the tumour will be attended with pulfation; but when from this caufe it cannot be made to fubfide by preffure, yet it is not to be fuppofed harmlefs, as in fact it requires to be treated with great caution. If the pulfations fhould arife from the real impulfe of the blood, it will be fhown by the increafe of the tumour,*" &c.

Not to detain y u any longer on a fubject which can be no further interefting than as it leads to a more exact defcription of the cafe, I fhall offer one important enquiry on this manner of treating aneurifms.

The only cafe on record, treated in this manner, is that related by Mr. Home. † As the aneurifm did not arife from injury the fac was confequently entire, which is no inconfiderable advantage under fuch a mode of cure. For if the blood is once coagulated in the fac, and in the portion of artery below it, the cure muft follow; becaufe the fac being impervious can only be diftended to a certain degree; after which the flow of blood, lower than the fuperior branches of the artery, would be effectually prevented. Hence we might expect thofe appear-

* Hunter on the blood, Page 196.

† Memoirs, &c. vol. I.

 ances ances defcribed by Mr. Home.—" The tumour," he obferves, " increafed to a confiderable fize; a great degree of fwelling and inflammation took place in the fac, and common integuments and mortification appeared to be coming on the fkin. While in this ftate no pulfation could be felt in the tumour, or the artery immediately above it, fo that the fteps preceding mortification had taken place, which put a ftop to the dilatation of the fac and all its confequences."

Though no one is more ready to admit that harmony of action, which Mr. Hunter has fo beautifully traced in many parts of the œconomy; yet, in the prefent inftance, Mr. Home feems to me to have prefumed upon it where it cannot be proved, and where it is altogether unneceffary. To account for the coagulation of blood in arteries above a mortified part, we need only refer to the fame fource as Mr. Home quotes. " There is," fays Mr. Hunter, " an important difference between death from mortification, and any other caufe. A dead man, or a limb cut from a living man, may be injected; but in a mortified part we can neither difcover blood veffels, mufcles, or cellular membrane. The whole has one uniform white leathery appearance."* It is true the fame phyfiologift obferves, that the coagulation of the blood, in fome cafes, is fo much higher than the mortification has extended, that it can only arife from a preparatory ftep to mortification itfelf.† But it does not appear probable that the difpofition to mortification fhould extend fo far as to bring the neighbouring veffels into action, and yet ceafe without mortification actually following. In the cafe from Mr. Hunter, above referred to, the patient died before the difeafe could extend fo far. We have other inftances of a preparatory action marking the future progrefs of a difeafe; but, I believe, in all fuch cafes, the difeafe itfelf never fails to follow.

This has been accurately afcertained by Mr. Hunter in cafes of morbid poifon, even when a difeafe has been fufpended for a time by a remedy; but it may be traced in moft of the commoneft operations of difeafe and reftoration. I fhall inftance only one. When matter has formed in any part, however diftant from the furface, we are for fome time in doubt whether it will make its way to the fkin, or be abforbed; but from the time that we fee a florid appearance become permanent on the fkin, we are fatisfied that the matter, however diftant, will make its way outward. If, afterwards, we perceive one part of this reddened fkin fomewhat elongated, and paler than the reft,

* Hunter's Lectures.—This quotation is from memory.

† Treatife on the Blood, &c. page 23.

reft, fo as to point, as we ufually term it, we are not lefs certain that the matter will find its firft exit at that place; and however inconvenient fuch a part may be, no endeavours of the furgeon can fruftrate the intentions of Nature. Should he even make an artificial opening, the part which pointed will ulcerate as deep as the abfcefs, though the contained matter may have been previoufly evacuated.

From this, and many other inftances of the kind, it is probable that the difpofition to mortification could not have extended fo far as to bring the neighbouring veffels into a preparatory action, without mortification itfelf following. It is indeed difficult to conceive how a part highly inflamed can fail to mortify when fuddenly deprived of all the blood that fed it; for this coagulation muft have extended to all the minuteft branches connected with the tumour, or prove unequal to the intention of Nature.

It feems therefore moft probable that the blood had coagulated in the fac, and probably in the artery between the fac and the place of preffure. In either cafe, the artery would contract itfelf, from the blood no longer paffing through it, or being much diminifhed in quantity. But the conftant efforts of the heart and larger arteries might for an inftant overcome this preffure in a variety of ways, in which cafe the blood fuddenly entering the contracted artery, would produce pain, either from the fudden change in the fituation of the artery, from the neighbouring nerve being affected, or from the blood flowing into the now impervious fac.

There are many reafons why the artery above the fac fhould recover itfelf, while the fac remains impervious. The firft is, that the fac has loft its elafticity. The artery, on the contrary, having contracted upon its contents, is likely to preferve a fmall diameter. If this is filled with coagulum, and the preffure is overcome before that coagulum is firm, or has even taken veffels, the coagulum may readily be forced into the fac and the blood follow, not only becaufe the diameter of the artery may be increafed, but becaufe as in this part the artery fends off few or no branches, its conical figure is with its apex towards the heart.* At the fame time, the fituation of the artery below the fac is very different in this refpect, and alfo in the obftruction which the blood muft meet with in paffing through the fac, now ftuffed with coagulum and deprived of its elafticity.

I fhould not have dwelt fo long on this fubject had it been merely

* Hunter on the blood, p. 168, & feq.

merely a matter of curiosity; but if, as the most exact and continued observation convinces me, in the case I have related, the pain during the pressure arose from the artery overcoming that pressure, it is obvious that the remedy is to increase it; and the success I met with justifies such a conclusion.

The advantages an operating surgeon may derive from such a fact appear to me very numerous. In cases of aneurism the whole operation of cutting, searching for, and tying the artery, which, how much soever simplified by Mr. Hunter's important improvement, is by no means trifling, may be saved. In cases of bleeding stumps, instead of the uncertain process of tying the artery higher, or even renewing the operation, such an application would promise every success, and be attended with few inconveniences. Since the late improvement in amputation by Mr. Allanson, the only thing which prevents an immediate union of the parts composing the stump is the ligature.

In warm climates the ligature proves the principal danger, from the frequent recurrence of locked-jaw. To lessen this danger, and the great pain that attends the common mode of tying the artery, it has been proposed to dissect the nerve from it. In the few cases that I have seen, where this has been attempted, it has been a matter of doubt with me whether the pain has been much lessened. At the same time the operation has been prolonged, and I have had reason to believe that the artery, thus deprived of the vessels that supported it,* has sloughed sooner, so as to endanger a bleeding stump.

If such a compressing instrument were applied over the artery of a limb to be amputated, some time before the operation, the surgeon would have known how far he might depend on its having completely stopped the flow of blood, and might perform his operation without embarrassment to himself, almost without uneasiness to his patient, and with the fairest prospect of success. For this purpose, the surgeon or his assistant, should remain with the patient, and if necessary increase the pressure, till he is satisfied there is no longer any danger of the artery recovering itself. The instrument might be left on after the operation is completed.

But most of all, in those unhappy cases of hæmorrhage from wounds, where the surgeon is absent, or called to a variety of cases at the same time, how much preferable would such an instrument be to the tourniquet? Should the wounded vessel be deep seated, or the hæmorrhage proceed from a number of wounded

* The vasa arteriarum are supplied not from the artery itself, but from the neighbouring vessels. Hunter on the Blood, &c. p. 131.

wounded ones, how dreadful and often how unfuccefsful does the attempt prove of taking up the artery. All this, however, and much more, will occur to you. Inftead, therefore, of proceeding any further, I ought to afk pardon for having detained you fo long. I cannot however omit one little incident, to fhow ftill further how little we need be afraid of the capacity of the veffels to perform their reftorative functions, after the obliteration of the large artery. Whilft the fore-finger was incapable of fenfe or motion, it was fcalded with boiling water from a tea-urn. Of this the patient knew nothing but by the evidence of his fight. Such was the cafe during the vefication, ulceration, and confequent healing of the part, which took place in as fhort a time as if the finger had received its ufual portion of blood.

